# Clinical characteristics of the autumn-winter type scrub typhus cases in south of Shandong province, northern China

**DOI:** 10.1186/1471-2334-9-82

**Published:** 2009-06-04

**Authors:** Yun-Xi Liu, Dan Feng, Ji-Jiang Suo, Yu-Bin Xing, Gang Liu, Li-Hua Liu, Hong-Ju Xiao, Ning Jia, Yan Gao, Hong Yang, Shu-Qing Zuo, Pan-He Zhang, Zhong-Tang Zhao, Jing-Si Min, Pei-Tian Feng, Shu-Bin Ma, Song Liang, Wu-Chun Cao

**Affiliations:** 1 Department of Nosocomial Infection Management and Disease Control, Institute of Hospital Management, Chinese People's Liberation Army General Hospital, Chinese People's Liberation Army Postgraduate Medical School, Beijing 100853, PR China; 2 Department of Health Statistics, Institute of Hospital Management, Chinese People's Liberation Army General Hospital, Chinese People's Liberation Army Postgraduate Medical School, Beijing 100853, PR China; 3 Department of Febrile Disease, Chinese People's Liberation Army General Hospital, Chinese People's Liberation Army Postgraduate Medical School, Beijing 100853, PR China; 4 State Key Laboratory of Pathogen and Biosecurity, Beijing Institute of Microbiology and Epidemiology, Beijing 100071, PR China; 5 School of Public Health, Shandong University, Jinan 250012, PR China; 6 Department of Internal Medicine, Wanggou Township Hospital, Feixian County, Shandong Province 273408, PR China; 7 Department of Internal Medicine, Shangye Township Hospital, Feixian County, Shandong Province 273401, PR China; 8 Department of Internal Medicine, Fangcheng Township Hospital, Feixian County, Shandong Province 273409, PR China; 9 College of Public Health, The Ohio State University, Columbus, Ohio 43210, USA

## Abstract

**Background:**

Before 1986, scrub typhus was only found endemic in southern China. Because human infections typically occur in the summer, it is called "summer type". During the autumn-winter period of 1986, a new type of scrub typhus was identified in Shandong and northern Jiangsu province of northern China. This newly recognized scrub typhus was subsequently reported in many areas of northern China and was then called "autumn-winter type". However, clinical characteristics of associated cases have not been reported.

**Methods:**

From 1995 to 2006, all suspected scrub typhus cases in five township hospitals of Feixian county, Shandong province were enrolled. Indirect immunofluorescent assay (IFA) was used as confirmatory serodiagnosis test. Polymerase chain reaction (PCR) connected with restriction fragment length polymorphism (RFLP) and sequence analyses were used for genotyping of *O. tsutsugamushi *DNAs. Clinical symptoms and demography of confirmed cases were analyzed.

**Results:**

A total of 480 scrub typhus cases were confirmed. The cases occurred every year exclusively between September and December with a peak occurrence in October. The case numbers were relatively higher in 1995, 1996, 1997, and 2000 than in other years. 57.9% of cases were in the group aged 21–50. More cases occurred in male (56%) than in female (44%). The predominant occupational group of the cases was farmers (85.0%). Farm work was reported the primary exposure to infection in 67.7% of cases. Fever, rash, and eschar were observed in 100.0%, 90.4%, and 88.5% of cases, respectively. Eschars formed frequently on or around umbilicus, abdomen areas, and front and back of waist (34.1%) in both genders. Normal results were observed in 88.7% (WBC counts), 84.5% (PLT counts), and 89.7% (RBC counts) of cases, respectively. Observations from the five hospitals were compared and no significant differences were found.

**Conclusion:**

The autumn-winter type scrub typhus in northern China occurred exclusively from September to December with a peak occurrence in October, which was different from the summer type in southern China. In comparison with the summer type, complications associated with autumn-winter type scrub typhus were less severe, and abnormalities of routine hematological parameters were less obvious.

## Background

Scrub typhus is a rickettsial disease caused by *Orientia tsutsugamushi *[1], which is transmitted to humans through infected chigger mites. Scrub typhus is widely distributed in Southeast Asia and the Pacific Rim including China [1,2]. When the rickettsia is transmitted through the bite of an infected mite to human, it begins to proliferate at the bite site and a characteristic skin lesion, known as an eschar, is formed. The pathogen then spreads systemically via the hematogenous and lymphogenous routes. Infected people develop various systemic symptoms and reactions including fever, cutaneous rash, lympadenopathy, elevations of C-reacting protein (CRP) and liver enzymes [2-4].

Prior to 1986, scrub typhus was only found endemic in southern China (south of the Yangtse River, or to the south of 31° north latitude), including 11 provinces (e.g., Guangdong, Hainan, Guangxi, Fujian, Zhejiang, Yunnan, Hunan province). Because human infections typically occur between March and November with a peak occurrence between June and August in the summer [5-7], so the scrub typhus is also called "summer type" scrub typhus, which is transmitted by the *Leptotrombidium deliense *mite [5-8]. The reservoir hosts are rodents mainly including *Rattus losea*, *R. flavipectus*, and *Apodemus agrarius *[5-7]. Major serotypes of summer type scrub typhus in many areas of southern China were Karp, Gilliam, and Kato types [5-7,9-11]. In China, genotypes of scrub typhus have not been systematically studied until recently [6,7,9]. However, the genotyping results obtained in Guangdong, Fujian, Hainan province of southern China revealed that Karp types were the key genotypes of summer type scrub typhus in these areas [12-14]. The summer type scrub typhus is caused by a relatively more virulent strain of *O. tsutsugamushi *[5,7]. Human cases caused by the summer type scrub typhus have common clinical features including fever, cutaneous rash, eschar and local lympadenopathy, and the associated complications were typically severe [5-7].

During the autumn-winter period of 1986, some residents in Mengyin county, south of Shandong province [15], and Dongtai, northern Jiangsu province [16] (both located in north of the Yangtse River, or to the north of 31° north latitude) developed an unknown fever, which was later identified to be caused by scrub typhus. As cases associated with this type of scrub typhus occurred from September to December with an occurrence peak in October, it was called "autumn-winter type". This type of scrub typhus was subsequently reported in many regions of northern China including Tianjing, Shanxi province, Hebei province, and Henan province [5-7,17,18]. The autumn-winter type scrub typhus is caused by a less virulent strain of *O. tsutsugamushi *and transmitted by *L. scutellare *mite [5-7,19]. The reservoir hosts are *A. agrarius*, *Cricetulus triton*, and *R. norvegicus *[5-7,19]. Although Gilliam types were identified by IFA as the key serotypes of autumn-winter type scrub typhus in many areas of northern China [5-7,14,19], the genotyping results acquired in Shandong, northern Jiangsu province in northern China showed that Kawasaki types were the key genotypes of this type of scrub typhus in the study areas (the genotypes in other areas were not studied or undetermined) [14,20-22]. Since 1986, people infected with autumn-winter type scrub typhus have been increasing in many areas of northern China [5-7,17,18]. However, clinical characteristics of the newly recognized scrub typhus have not been studied. In this study, we summarize the clinical characteristics of human infections by autumn-winter type scrub typhus in Feixian county, south of Shandong province, northern China.

## Methods

### The study site

**Figure 1:**
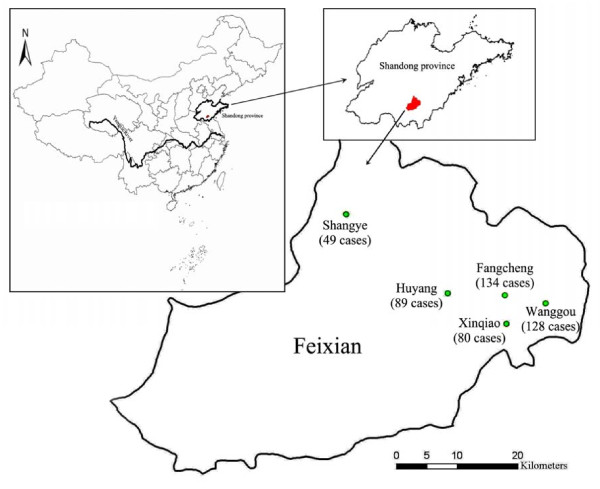
**Distribution of the study sites**. The map shows location of the study sites and distribution of confirmed cases in the five towns of Feixian county in southern Shandong province, China. The numbers in parentheses denote the total number of cases in each town during the 12-year study period.

The study was conducted from 1995 to 2006 in Feixian county (117°11'–118°18'E, 35°01'–35°33'N), which is located in a mountainous area in the south of Shandong province (Figure 1). The northwestern parts of Feixian county belong to the range of Meng Mountains, while the south parts belong to the range of Ni Mountains respectively. The northeastern and central parts of Feixian county are flatlands in front of mountain within its boundaries, and located in temperate and sub-humid areas with distinct four seasons, the county has an annual average temperature 13.4°C with the highest average 26.3°C in July and lowest -1.8°C in January [23]. We selected five coadjacent township hospitals (Fangcheng, Wanggou, Huyang, Xinqiao, and Shangye), which serve for about 260,000 local residents in the surrounding 22 km^2 ^area in the northeast of Feixian county, where scrub typhus was first reported in 1988 and became endemic since then during the autumn-winter seasons [5-7].

### Criteria for case selections

Between 1995 and 2006, patients who were admitted to the five township hospitals, had acute undifferentiated fever and clinical manifestations indicative of scrub typhus were enrolled in the study as suspected cases. The clinical manifestations included acute fever, eschars, local lympadenopathy, and maculopapular rashes on the trunk and proximal limbs. For each enrolled case, information on past activities (e.g., farming, playing, and recreation in the farm field where rodents were present) within 1–3 weeks (this time window is the incubation period of the autumn-winter type scrub typhus) prior to his/her illness were recorded. After informed consent was obtained, acute and convalescent blood samples and eschars from suspected cases were collected for subsequent IFA and PCR analyses. People with a history of known chronic medical problems or illness were excluded in the study. The study was approved by the Ethical and Scientific Review Subcommittee of the Ministry of Public Health of China.

### Confirmatory diagnosis

For each suspected case, the serum was assayed by IFA for detecting the IgM and IgG antibodies against pooled Karp, Kato, and Gilliam strains of *O. tsutsugamushi *antigens [5,7,14,19]. Serotyping of each positive serum was carried out as previously described [5,7,14,22]. A confirmed scrub typhus case was defined as (1) IgG titer ≥ 4-fold increase in paired serum specimens; and (2) IgM titer ≥ 1:80 or IgG titer ≥ 1:400 in a single serum sample, [5,24-26]. PCR method became available in 2001 and was used for detection and genotyping of *O. tsutsugamushi *connected with RFLP and nucleotide sequences analyses since then [21,22,27].

### Data collection

Questionnaire we developed were administered by physicians working in the five township hospitals. The questionnaire were designed to include (1) demography: age, sex, education level, and occupation; (2) epidemiologic data: symptom onset date, residence place, and activities possibly associated with scrub typhus infection within 1–3 weeks prior to onset of symptoms; (3) clinical signs and symptoms: fever, eschar, rash, lymphadenopathy, and others; (4) routine hematological examination: white blood cell (WBC) counts, platelet (PLT) counts, red blood cell (RBC), hemoglobin (HGB) and hematocrit (HCT); and (5) diagnosis: results of IFA and PCR analyses. All collected data were double-entered in spreadsheet for subsequent statistical analyses.

### Statistical analyses

Statistical analyses were performed using the Chi-square test (or Fisher's exact test) for categorical data. A difference was considered statistically significant if the *P *value was less than 0.05. Ranges were indicated by IQR (interquartile range).

## Results

### Confirmed cases of scrub typhus

From 1995 to 2006, a total of 678 suspected cases were enrolled in the study. Based on results from IFA examination, 280 scrub typhus cases were confirmed based on a fourfold or greater increase between paired serum specimens; 86 cases were confirmed by having an IgM titer ≥ 1:80 in a single serum sample; and 114 cases were confirmed by having an IgG titer ≥ 1:400 in a single serum sample. The serotypes of the 480 IFA-positive samples were all Gilliam types. The rest 198 cases were non-scrub typhus cases as identified by IFA. 15 isolates were obtained from 40 IFA-positive cases during the study period [19,21,22]. Figure 1 showed location of the study area and distribution of the 480 cases in the five towns: 134 (27.9%) cases in Fangcheng, 128 (26.7%) cases in Wanggou, 89 (18.5%) cases in Huyang, 80 (16.7%) cases in Xinqiao, and 49 (10.2%) cases in Shangye.

Among the IFA-positive samples collected from 2001 to 2006, 34 out of the 45 acute stage blood samples of cases were PCR-positive. Ten eschars from 10 cases whose serum samples were IFA-positive and blood samples were PCR-positive were also tested positive by PCR. In addition, 15 isolates were tested positive by PCR [21,22]. The genotypes of the above 59 PCR-positive samples were similar to Kawasaki strain with one mutation of restriction endonuclease *Hha*-site (details were discussed elsewhere, see references 21,22,27).

Chloramphenicol was administered orally at dosages of 1.5–2.5 g 4×/day for 4–5 days for adult cases and 25–50 mg/kg 4×/day for 4 to 5 days for pediatric cases, respectively. The median interval to defervescenct-period after chloramphenicol therapy was 2 days (range 1–5 d). All of the cases defervesced after treatment, except one case (male, 70-year-old) who was initially misdiagnosed by the country infirmary. The case was sent to Shangye township hospital with serious multiple system/organ failures, and eventually died after all efforts for rescuing him failed.

### Seasonal distribution of confirmed cases

In the study areas over the past 12 years, scrub typhus cases occurred exclusively every year from the period of day 11–20, September to the period of day 1–10, December, and seasonal distributions of the cases were similar from 1995 to 2006 (Table 1). During the study period, no case was reported before September and after December and peaks of cases occurrences were all in October, followed by a sharp drop in case numbers. The case numbers were relatively higher in 1995, 1996, 1997, and 2000 than in other years.

**Table 1:** Yearly and seasonal distribution of confirmed cases

Year	September			October			November			December			Total (%)
					
	1–10	11–20	21–30	1–10	11–20	21–31	1–10	11–20	21–30	1–10	11–20	21–31	
1995	0	0	1	10	44	41	19	4	0	0	0	0	119 (24.8)
1996	0	0	3	4	8	20	12	10	1	0	0	0	58 (12.1)
1997	0	0	3	8	20	10	7	2	0	0	0	0	50 (10.4)
1998	0	0	0	6	2	19	5	0	0	0	0	0	32 (6.7)
1999	0	0	0	7	11	6	5	0	0	0	0	0	29 (6.0)
2000	0	0	0	8	15	5	7	5	0	0	0	0	40 (8.3)
2001	0	0	0	8	10	2	1	0	0	0	0	0	21 (4.4)
2002	0	1	2	9	13	3	3	4	0	0	0	0	35 (7.3)
2003	0	1	0	2	3	7	2	0	0	1	0	0	16 (3.3)
2004	0	0	2	0	11	5	5	1	0	0	0	0	24 (5.0)
2005	0	0	1	5	4	16	6	0	1	0	0	0	33 (6.9)
2006	0	0	0	1	6	11	4	0	1	0	0	0	23 (4.8)
Total	0	2	12	68	147	145	76	26	3	1	0	0	480
(%)	0	(0.4)	(2.5)	(14.2)	(30.6)	(30.2)	(15.8)	(5.4)	(0.6)	(0.2)	(0)	(0)	(100)

### Sex and age distributions of confirmed cases

Of 480 confirmed cases, 56% (269/480) were male patients and 44% (211/480) were female, respectively. The percentage of male cases was higher than that of females in all groups except for the group aged 31–40 (χ^2 ^= 42.4, P < 0.001). 278 (57.9%) cases were in the group aged 21–50, followed by 102 (21.3%), 79 (16.5%) and 21 (4.4%) cases in the group aged 51–70, 0–20, and ≥ 71, respectively. The youngest and oldest cases were 1.5 and 81 years old, respectively (Table 2).

**Table 2:** Age and occupational distributions and activities (1–3 weeks prior to symptom appearance) of confirmed cases

		Cases (%)	(Male: Female, %)
Age (Years)			
	0–10	46 (9.6)	(76:24)
	11–20	33 (6.9)	(79:21)
	21–30	78 (16.3)	(51:49)
	31–40	99 (20.6)	(46:54)
	41–50	101 (21.0)	(56:44)
	51–60	47 (9.8)	(55:45)
	61–70	55 (11.5)	(53:47)
	≥ 71	21 (4.4)	(52:48)
Occupation			
	Children at preschool age	20 (4.2)	(75:25)
	Pupils	50 (10.4)	(78:22)
	Farmers	408 (85.0)	(52:48)
	Others	2 (0.4)	(50:50)
Activities			
	Farm work	325 (67.7)	(53:47)
	Play on grassland	65 (13.5)	(75:25)
	Recreation	20 (4.2)	(65:35)
	Housework	25 (5.2)	(8:92)
	Unknown	45 (9.4)	(71:29)

### Occupation and activities possibly connected with infection

The predominant occupational group of the cases was farmers (85.0%), followed by pupils (10.4%), and preschool children (4.2%). The percentage of male cases was higher than that of females in the above three groups (χ^2 ^= 28.4, *P *< 0.001). The human activities possibly associated with infection included farm work (325 cases, 67.7%), playing on grassland (65 cases, 13.5%), recreation (20 cases, 4.2%), and housework (25 cases, 5.2%). The percentage of male cases was higher than that of females in all categories except the housework group (χ^2 ^= 119.6, *P *< 0.001, Table 2).

### Clinical symptoms

Table 3 summarize the symptoms and signs of all 480 confirmed cases at the time of admission to hospitals with additional 274 cases previously collected from 1996 to 2005 in Shaoguan, Guangdong province, southern China [28], where the "summer type" scrub typhus was endemic. In present study, cases manifested with the common clinical symptoms of scrub typhus: fever (100.0% of confirmed cases), rash (90.4%), eschar (88.5%), and regional lymphadenopathy (60.6%). Headache (100.0%), myalgia and prostration (100.0%), loss of appetite (82.9%), chills (67.3%), abdominal pain (48.8%), erythematous flushes (48.8%), nausea/vomiting (43.8%), retro-orbital pain (17.3%), and flank tenderness (10.0%) were observed respectively. Hepatosplenomegaly and electrocardiogram abnormity were reported in 84 (17.5%) and 38 (7.9%) cases, respectively. No cases had the severe complications such as toxic myocarditis, alimentary tract hemorrhage, pleural fluid, or abdominal dropsy which were reported in southern China [28].

**Table 3:** Clinical characteristics of confirmed cases at the time of hospital admission

Clinical symptoms		Present study N = 480 (%)	**Other study **[28] N = 274 (%)	*P*-value
Typical symptoms of scrub typhus				
	Fever	480(100.0)	274(100.0)	--
	Maculopapular rashes appeared on the trunk and proximal limbs	434 (90.4)	102(37.2)	< 0.001^a^
	Skin eschars	425 (88.5)	240(87.6)	0.697 ^a^
	Regional lymphadenopathy	291 (60.6)	248(90.5)	< 0.001^a^
General symptoms of febrile disease				
	Chills	323 (67.3)	125(45.6)	< 0.001^a^
	Loss of appetite	398 (82.9)	243(88.7)	0.033^a^
	Erythematous flushes on the face, neck and upper bosom	234 (48.8)	--	--
	Myalgia and prostration	480 (100.0)	250(91.2)	< 0.001^a^
Ache				
	Headache	480 (100.0)	135(49.3)	< 0.001^b^
	Retro-orbital pain	83 (17.3)	188(68.6)	< 0.001^a^
Heart damage				
	Electrocardiogram abnormity	38 (7.9)	74(27.0)	< 0.001^a^
	Toxic myocarditis	0	57(20.8)	< 0.001^b^
Digestive system damage				
	Abdominal pain	234 (48.8)	67(24.5)	< 0.001^b^
	Nausea/vomiting	210 (43.8)	62(22.6)	< 0.001^a^
	Hepatosplenomegaly	84 (17.5)	166(60.6)	< 0.001^b^
	Alimentary tract hemorrhage	0	34(12.4)	< 0.001^b^
	Pleural fluid, or abdominal dropsy	0	27(9.9)	< 0.001^b^
Kidney damage				
	Flank tenderness	48 (10.0)	86(31.39)	< 0.001 ^a^

**a **– Chi-square test; **b **– Fisher's exact test.

### Location of eschars

Of 480 confirmed scrub typhus cases, 88.5% (425/480) exhibited one eschar, of which 89.2% (240/269) was found in male while 87.7% (185/211) was observed in female patients. None of confirmed cases showed two or more eschars (Table 4).

**Table 4:** Eschar distributions on bodies of confirmed scrub typhus cases

Eschar location	Total cases (%)	Male cases (%)	Female cases (%)	*P*-value
Head, face, eye, and neck	14 (3.3)	9 (3.8)	5 (2.7)	0.549 ^a^
Front chest above umbilicus (including breast)	40 (9.4)	10 (4.2)	30 (16.2)	<0.001^a^
Back	11 (2.6)	8 (3.3)	3 (1.6)	0.362 ^b^
Axilla	89 (20.9)	49 (20.4)	40 (21.6)	0.762^a^
On or around umbilicus, abdomen areas, and front and back of waist	145 (34.1)	86 (35.8)	59 (31.9)	0.395^a^
Perineal, inguinal areas, anus, buttock, penis or scrotum/labia	88 (20.7)	54 (22.5)	34 (18.4)	0.298^a^
Upper extremities	18 (4.2)	11 (4.6)	7 (3.8)	0.685^a^
Lower extremities	20 (4.7)	13 (5.4)	7 (3.8)	0.431^a^
**Total**	**425 (100.0)**	**240 (100.0)**	**185 (100.0)**	

**a **– Chi-square test; **b **– Fisher's exact test.

Eschars formed frequently on or around umbilicus, abdomen areas, and front and back of waist (34.1%), followed by axilla (20.9%), and perineal, inguinal areas, anus, buttock, penis or scrotum/labia (20.7%). There were no differences in the locations of the eschar sites between the sexes, except that the eschar appeared more frequently on the front chest above the umbilicus of the females (16.2%) than males (4.2%) (χ^2 ^= 17.8, *P *< 0.001).

### Routine hematological examinations

For routine hematological examinations of the confirmed cases at the time of admission, both normal and abnormal (e.g. increased or decreased counts) hematological parameters were recorded. Normal results were observed in 88.7% (WBC counts), 84.5% (PLT counts), 89.7% (RBC counts), 68.8% (HGB), and 43.9% (HCT) of cases, respectively, while 10.5%, 10.9%, 8.4%, 12.2%, and 15.1% of cases showed increased counts for each of these categories, respectively (Table 5).

**Table 5:** Routine laboratory findings of confirmed cases at the time of hospital admission to hospitals

Findings	Median (IQR)	Present study N = 478 (%)	**Other study **[28] N = 274 (%)	*P*-value
WBC counts (10^9^/L)	6.7 (5.4–9.3)			
Increase (> 10.0)		50 (10.5%)	90 (32.9)	<0.001^a^
Normal (4.0–10.0)		424 (88.7%)	110 (40.1)	
Decrease (< 4.0)		4 (0.8%)	74 (27.0)	
PLT counts(10^9^/L)	143 (120–206)			
Increase (>300)		52 (10.9%)	---	<0.001^a^
Normal (100–300)		404 (84.5%)	134 (48.9)*	
Decrease (<100)		22 (4.6%)	140 (51.1)	
RBC counts (10^12^/L)	5.1 (4.4–5.3)			
Increase (>5.5)		40 (8.4%)	---	
Normal (3.5–5.5)		429 (89.7%)		
Decrease (<3.5)		9 (1.9%)		
HGB (g/L)	149 (119–151)			
Increase (>160)		58 (12.2%)	---	
Normal (110–160)		329 (68.8%)	---	
Decrease (<110)		91 (19.0%)		
HCT (L/L)	0.4 (0.3–0.5)			
Increase (>0.50)		72 (15.1%)	---	
Normal (0.4–0.50)		210 (43.9%)	---	
Decrease (<0.4)		196 (41.0%)	---	

* The number included cases with increased and normal PLT counts (They were not differentiated in the report).

**a **– Chi-square test.

## Discussion

In this study, we documented occurrence periods for the autumn-winter type scrub typhus cases. The seasonal variation of scrub typhus cases in northern China was similar to that reported in Korea, where human cases of scrub typhus were reported to increase in October and peak in November [29]. This pattern was, however, different from those reported in Japan [30,31] and in southern China [5-7]. In Japan, a bimodal pattern of occurrence of cases – one in spring and another in autumn-winter (the latter being similar to the autumn-winter type reported in current study) [30,31] – was reported. Yet in southern China, scrub typhus, caused by the summer type, was endemic with a single peak of infections occurring in summer [5-7,28]. Because humans are infected through bites of the larva of the chigger mites, seasonal variations in scrub typhus infections may be in part due to seasonal fluctuations of the larval chigger mites as well as their rodent hosts. In Japan, the bimodal pattern of occurrence of cases was reported to relate to population dynamics of the two different species of chigger mites. Cases occurred in the autumn-winter period in many areas of Japan were mainly due to *L. scutellare*, while cases in the spring period in western Japan were caused primarily through *L. pallidum *[30,31]. In southern China, seasonal variations in scrub typhus infections were commonly consistent with seasonal variations in population of *L. deliense *[5-8]. The authors conducted a survey on rodent dynamics between May 1995 and April 1996 in the same study area and found that the population abundance of *A. agrarius*, the most abundant rodent species in the enzootic area, showed a bimodal pattern in a year – the first in July and the second in October. *O. tsutsugamushi *was isolated from *A. agrarius*, which was confirmed as the host for scrub typhus in the areas [19,32]. Seasonal variations in chigger mites on the field rodents were also observed in this one-year study, showing that *L. scutellare *first appeared during the period of day 11–20 of September (the chigger index was 13.51) and its abundance then increased sharply in October (the chigger index increased to 22.68). The chigger population peaked during the period of day 11–20 of November (the chigger index peaked to 36.56) and dropped substantially in December (the chigger index in December was 0.47). In addition, *O. tsutsugamushi *was also isolated from *L. scutellare*, which was confirmed as the vector of scrub typhus in the study area [19,33]. The present study, together with our previous work [19,32,33], suggests that the time when rodents cause maximum larval chigger infestation overlaps the period when the highest incidence of scrub typhus infections were reported in humans. Based on previous reports and this study, the main differences between the "summer type" and "autumn-winter type" scrub typhus in China were summarized in Table 6.

**Table 6:** Key differences between "summer type" and "autumn-winter type" scrub typhus in China

	The summer type	The autumn-winter type	References
Seasonal distribution of human cases	Between March and November with a peak occurrence in the summer between June and August	Exclusively from September to December with a peak occurrence in October	[5-7]
Geographical distribution	Endemic in southern China (south of the Yangtse River), including Guangdong, Hainan, Guangxi, Fujian, Zhejiang, Yunnan, and Hunan province.	Endemic in northern China (north of the Yangtse River), including Shandong, northern Jiangsu, Tianjing, Shanxi, Hebei, and Henan province	[5-7]
Key reservoir hosts	*Rattus losea, R. flavipectus*, and *Apodemus agrarius*	*A. agrarius, Cricetulus triton*, and *R. norvegicus*.	[6,7]
Key vector chigger mites	*Leptotrombidium deliense*	*L. scutellare*	[5-8]
Virulence of *O. tsutsugamushi isolates*	More virulent, because: (1) Being tested with mice, the median lethal dose (LD_50_) values of *O. tsutsugamushi *isolates were 10^-5 ^– 10^-8^; (2) *O. tsutsugamushi *could be successfully isolated from cases by normal experimental mice without any treatment	Less virulent, because: (1) Partial *O. tsutsugamushi *isolates do not lead to death of mice; (2) Being tested with mice, LD_50 _values of most *O. tsutsugamushi *isolates were 10^-0.5 ^– 10^-3^; (3) In order to isolate *O. tsutsugamushi *successfully from cases, the experimental mice must be treated by cyclophosphamide to suppress immunity	[5,7]
Key serotypes	Karp type, Gilliam type, and Kato type	Gilliam type	[5-7,9-11,14,19]
Key genotypes*	Karp type	Kawasaki type	[12-14,20-22]
Clinical features and complications	Cases have the common clinical features of scrub typhus such as fever, cutaneous rash, eschar and local lympadenopathy. However, the associated complications of this type scrub typhus were severe	Cases also have the common clinical features of scrub typhus such as fever, cutaneous rash, eschar and local lympadenopathy. However, the associated complications of this type of scrub typhus were less severe than those of the summer type	[5-7,15-18,28]

* **Note: **In China, the genotypes of scrub typhus have not been systematically studied until now. Data about genotypes of scrub typhus provided in the Table were only based on the primary results obtained in a few of areas in China. Karp types were identified as the key genotypes of the summer scrub typhus in Guangdong, Fujian, Hainan province in southern China [12-14], while Kawasaki types were identified as the key genotypes of the autumn-winter type scrub typhus in Shandong, northern Jiangsu province in northern China [14,20-22]

Although certain clinical symptoms occurred more frequently in autumn-winter type cases than in summer type cases, regional lymphadenopathy, retro-orbital pain, electrocardiogram abnormity, hepatosplenomegaly, and flank tenderness were less common in the autumn-winter type cases. In contrast, some severe complications such as toxic myocarditis, alimentary tract hemorrhage, pleural fluid, or abdominal dropsy, commonly reported in the summer type cases in southern China [28], were not found at all in present study (Table 4). In addition, the abnormalities of WBC and PLT counts in present study were also less obvious than those reported for the summer type. The reports in southern China suggested that the percentages of cases with the increased and decreased WBC counts were 32.9% and 27.0% respectively, which were higher than (χ^2 ^= 219.7, *P *< 0.001) those reported in current study. Thrombocytopenia was reported in 51.1% cases in southern China (Table 5) [28], also higher than (χ^2 ^= 222.7, *P *< 0.001) that obtained in current study.

The reported percentages of eschar formation showed substantial variations across different studies [34-37], which could be explained in part by disparity of physicians experiences. Eschars could be detected relatively frequently on white-skinned Japanese children, however, it is relatively difficult to detect eschars on dark-skinned Thai pediatric cases [35-37]. Previous studies from some new endemic areas in northern China showed that the percentages of scrub typhus cases with eschars were 15% [38], 34% [39], 84% [40] and 100% respectively [18]. In our study, 88.5% of cases had eschars. This is relatively high compared to other reports. Part of the reason is that physicians in our study team have worked there for many years and were very familiar with eschars. In addition, in our study we carried out thorough body examination for each suspected scrub typhus case, which may have contributed to high detection rate of eschars.

Irons et al (1947) reported that 45% of confirmed eschars in US army personnel were detected on the feet and legs. Perineum, inguinal area, and axilla were also the preferentially eschar-manifested areas [41]. Kim et al (2007) reported that among the 162 adult scrub typhus cases in southwestern area of Korea, most cases had eschars on the front of the body. Eschars were primarily detected in males within 30 cm below the umbilicus. Yet a different pattern was seen in females – the most prevalent area in females was the front chest above the umbilicus [42]. In present study, a similar distribution of eschar was seen for both males and females except that eschars were more frequently detected in front chest above umbilicus in females than in males (Table 4). The distribution of eschar on body surface might be associated with dressing styles and personal hygiene, as the two factors affect how and where chiggers entered and stayed on the body surface [5,6,9]. Ten eschars from 10 cases whose serum samples were IFA-positive and blood samples were PCR-positive were also tested positive by PCR. This result suggested that eschar could be used as an alternative indicator for the diagnosis of *O. tsutsugamushi *infection [27]. In addition, *O. tsutsugamushi *DNAs were detected in all blood samples collected from 10 cases with eschar, indicating that the cases were bitten by scrub typhus infected chiggers.

Serodiagnosis methods of scrub typhus include Weil-Felix test, IFA, enzyme-linked immunosorbent assay (ELISA) and indirect immunoperoxidase (IIP). Weil-Felix test is neither sensitive nor specific, and replaced by IFA [43,44]. IFA is a well method for serodiagnosis of scrub typhus if it was conducted by skilled lab-persons. However, it always provides false positive results. Therefore, the real-positives might be lower than that obtained by IFA. ELISA and IIP are more accurate than the IFA [45-47], but the two methods were not commercially available in China. Thus IFA was still widely used in China for serodiagnosis of scrub typhus until now. In present study, the confirmatory serodiagnosis of scrub typhus was made in case of a fourfold or greater rise in titers between paired acute and convalescent sera, or IgM or IgG titer in a single serum above 1:80, or 1:400 [5,24-26]. However, a few of cases whose IgM or IgG titer in a single serum under 1:80, or 1:400 might be neglected by IFA test according to this diagnosis criterion, especially when their convalescent sera were unavailable. This is a limitation of the present study.

The Kawasaki strain was first isolated from Japanese scrub typhus cases by using cyclophosphamide-treated mice in Miyazaki prefecture, and it was then found to be widely distributed in other infected areas of Japan, such as Kyushu, Kanagawa prefectures [48,49]. Kawasaki strain was defined as a new type of *O. tsutsugamushi *distinguishable from prototype strains Gilliam, Karp, and Kato by antigenic analyses using polyclonal and monoclonal antibodies [49]. However, some degree of cross-reactivity existed between the 54- to 56 kilodalton polypeptides of Kawasaki and Gilliam strains in immunoblotting analyses [50]. Ohashi et al [51] also found that the sequence homology of scrub typhus antigen 56-kilodalton (Sta56) surface protein gene between Kawasaki and Gilliam strain was the highest among the known *O. tsutsugamushi *strains, and thus high cross-immunoreaction existed between the two antigens. Because Kawasaki strain was not available as antigen in China, we had to use Karp, Kato, and Gilliam strains as antigens for IFA. In present study, serotypes of IFA-positive sera were found to be the Gilliam type, while genotyping results of eschars, and isolates from some serologically confirmed cases suggested that the genotype to be similar to the Kawasaki strain. This inconsistency could be in part due to the existence of cross-immunoreaction between Kawasaki and Gilliam strain [50,51], such that antibodies to Kawasaki strain in the sera of cases in northern China could also be tested positive by IFA when we used Gilliam strain as antigen instead of Kawasaki strain.

## Conclusion

The study, for the first time, comprehensively summarizes clinical and epidemiologic characteristics of the autumn-winter type scrub typhus in China. This type of scrub typhus emerged in 1986 in northern China and occurred exclusively from September to December with an occurrence peak in October, which was different from the summer type endemic in southern China. In comparison with the summer type, complications associated with autumn-winter type scrub typhus were less severe, and abnormalities of routine hematological parameters were less obvious. Despite some limitations inherent in the present study, this 12-year study provides important information on clinical and epidemiologic features of the autumn-winter type scrub typhus. These findings provide valuable information in assisting physicians in the diagnosis, treatment, and prevention of the disease.

## Competing interests

The authors declare that they have no competing interests.

## Authors' contributions

YL carried out the study design, epidemiological investigation, and drafted the manuscript. JS, YX, GL, LL, HX, NJ, YG, HY, SZ, PZ, JM, PF, SM participated in the epidemiological investigation, and sample and data collection in the field. DF, ZZ, WC and SL participated in the study design and helped to draft the manuscript. YL, SL, WC conceived the study and coordinated all the activities. All authors read and approved the final manuscript.

## Pre-publication history

The pre-publication history for this paper can be accessed here:

http://www.biomedcentral.com/1471-2334/9/82/prepub
